# Annual Plants and Thermoplastics in the Production of Polymer and Lignocellulose Boards

**DOI:** 10.3390/ma16124400

**Published:** 2023-06-15

**Authors:** Aleksandra Banaszak, Magdalena Woźniak, Dorota Dziurka, Radosław Mirski

**Affiliations:** 1 Department of Mechanical Wood Technology, Poznan University of Life Sciences, Wojska Polskiego 28, 60-627 Poznań, Poland; dorota.dziurka@up.poznan.pl (D.D.); radoslaw.mirski@up.poznan.pl (R.M.); 2 Department of Chemistry, Faculty of Forestry and Wood Technology, Poznan University of Life Sciences, Wojska Polskiego 75, 60-625 Poznań, Poland; magdalena.wozniak@up.poznan.pl

**Keywords:** polymer–straw boards, thermoplastic polymers, annual plants, mechanical properties

## Abstract

This study investigated the mechanical, physical, and thermal properties of three-layer particleboards produced from annual plant straws and three polymers: polypropylene (PP), high-density polyethylene (HDPE), and polylactic acid (PLA). The rape straw (*Brassica napus* L. var. Napus) was used as an internal layer, while rye (*Secale* L.) or triticale (*Triticosecale* Witt.) was applied as an external layer in the obtained particleboards. The boards were tested for their density, thickness swelling, static bending strength, modulus of elasticity, and thermal degradation characteristics. Moreover, the changes in the structure of composites were determined by infrared spectroscopy. Among the straw-based boards with the addition of tested polymers, satisfactory properties were obtained mainly using HDPE. In turn, the straw-based composites with PP were characterized by moderate properties, while PLA-containing boards did not show clearly favorable properties either in terms of the mechanical or physical features. The properties of straw–polymer boards produced based on triticale straw were slightly better than those of the rye-based boards, probably due to the geometry of the strands, which was more favorable for triticale straw. The obtained results indicated that annual plant fibers, mainly triticale, can be used as wood substitutes for the production of biocomposites. Moreover, the addition of polymers allows for the use of the obtained boards in conditions of increased humidity.

## 1. Introduction

Replacing the traditionally used particles in particleboard production with particles of annual crops is still a current topic. The reasons that wood particles are being substituted are, among others, the high prices, storage problems, intense market competition, and lack of suitable quality materials [1]. In turn, annual products are available in abundant volume throughout the world. It is worth highlighting that the forecast for annual surplus straw in Poland in 2025 is circa 14 thousand tons [2]. The most widely available straws in Poland are cereal straws (73.5%) and rape straws (9.1%) [3].

The literature describes the use of various lignocellulosic materials as a substitute for wood in particleboard manufacturing. The particleboards were made of wheat straw, rape straw, rice straw, corn straw, reed stalk chips, rye straw, triticale straw or groundnut shell, and rice husk wastes [4,5,6,7,8,9,10,11,12,13,14,15,16]. The data in the literature also showed that white mustard can be applied as a good alternative to wood in particleboard production [17]. Moreover, evening primrose straw, kiwi, grape, and tomato stalks and coffee or tea waste are interesting options in the replacement of wood chips in the manufacturing of particleboards [18,19,20,21]. Although many studies have been conducted on the use of various lignocellulosic materials for the production of oriented strand boards (OSBs), not all of the obtained materials provide the physical and mechanical properties required in construction (type five according to the EN 310 [22]). In our previous research, we analyzed the possibility of replacing pine chips with chips from annual plants (rye, triticale, rape, reed, and corn straw) in the outer layers of boards intended for the construction and furniture industry [11]. The use of annual plant straw was particularly favorable for the modulus of elasticity and smaller thickness swelling as compared with the pine boards. Moreover, the results indicated that, in specific conditions, all tested plants could serve as a partial substitute for wood chips in the external layers of particleboards [11]. Annual plants are an attractive alternative to wood chips in the production of chipboards due to, among other things, their low price, lower hygroscopicity, and specific gravity or better thermal and acoustic isolation [9,23,24,25]. On the other hand, straw has a larger amount of wax on the surface, which causes difficulties in using conventional adhesives. However, the modification of straws or adhesives such as pMDI (polymeric diphenylmethane diisocyanate) is the solution to this problem [6,26,27].

The particles of straw were also successfully used as a filler in a WPC (wood–plastic composite), using, among other straws, rape, sisal, hemp, jute fibers, and rice straw [28,29,30]. Mainly WPC is manufactured by methods used in the plastic industry. The exciting approach is flat pressing, which was used by numerous researchers [31,32,33,34,35,36,37]. The OSB or particleboard with polymer addition in inner layers was characterized by better physical properties, which maintained good mechanical properties. The plastic industry is very powerful, and the annual waste generated after using plastic products is still a big concern in our society. Using surplus straws and plastic polymers in particleboard is one idea to face the market’s needs and gain a fully valuable product.

In our previous research, we investigated the effect of various types of straw (rye, triticale, and rape) and various type of thermoplastics (HDPE, low-density polyethylene (LDPE), polystyrene (PS), and PP) on the properties of five-layer boards [31]. The results showed that the properties of the obtained boards strongly depended on both kinds of the polymer and straw used [31]. The research described by Mihai et al. [38,39] indicated that triticale straw has great potential in the production of thermoplastic composites. Moreover, the triticale content, the presence of maleic anhydride grafted polypropylene as a coupling agent, and the addition of calcium oxide as a reactive additive have an influence on the properties of PP/triticale straw biocomposites [38,39]. Moreover, biocomposites based on triticale straw and PLA were characterized by good properties, and due to their biodegradability of both PLA and triticale, they are more environmentally friendly than synthetics based on thermoplastics [40]. Moreover, the PP and rye-husk-based composites showed good performance compared to softwood composites, including better elongation at break and better Charpy impact strength [41].

As shown above, the application of straw from various annual plants, including triticale and rye, as a substitute for wood in the production of composites has been reported in the literature [30,31,32,33,34,35,36,37]. In this paper, the characterization of particleboards made from straw (triticale and rye) with the addition of three polymers, namely PP, HDPE, and PLA, is reported. To the best of the authors’ knowledge, this is the first report about the mechanical, physical, and thermal properties of three-layer polymer–straw boards produced based on triticale and rye and three different polymer matrices and with rape straw as an inner layer, with different moisture contents for the layers.

## 2. Materials and Methods

### 2.1. Materials

Three-layer boards were manufactured from particles of rape straw (*Brassica napus* L. var. Napus) as an internal layer and particles of cereal straw, i.e., triticale (*Triticosecale* Witt.) and rye (*Secale* L.), with a 30% addition of polymers as an external layer. The control boards were made without the addition of polymers. Polymers used as an addition in external layers were polypropylene (PP), high-density polyethylene (HDPE), and polylactic acid (PLA). PP and HDPE were obtained from bags for lunch and garbage, respectively. The bags are widely available at the most significant discounts in Poland. The bags were cut into thin strips 5 mm wide and 20–30 mm long. PLA was used in the form of granules. In Table 1, the structure of the boards is presented. The rye and the triticale straws were prepared traditionally, as described in detail in our previous work [11]. The very fine fraction passing through a sieve with a 0.5 × 0.5 mm^2^ mesh was removed. The straw particles prepared this way had a bulk density of 60 ± 2.5 kg/m^3^. A significant difference for the chosen straws was the width of the particles; i.e., wider and slightly thicker particles are characteristic of triticale [33]. To increase the compression of the middle layer, the rapeseed chopped strands were sieved through the sieve with a mesh of 2 × 2 mm^2^. This decreased the bulk density from about 80 kg/m^3^ to 48 ± 3.2 kg/m^3^. That was important because of the higher humidity of this layer. Due to adding polymers, the outer layers lost their initial low bulk density and vulnerability to compression during pressing.

### 2.2. Board Manufacturing

The chips were glued with the same amount (5% of dry weight) of pMDI (polymeric diphenylmethane diisocyanate, Ongronat^®^ 2100, BorsodChem Group, Kazincbarcika, Hungary) in all layers and all types of boards. Ongronat^®^ 2100 is a brown (20 °C, 1013 hPa) non-flammable liquid of a density of 3 g/cm^3^, viscosity of 210 mPa·s, contractual dry matter content of 100%, NCO content of 30.6%, and chlorine hydrolytic content of 127 ppm. Straws of annual plants show low gluability for commonly used formaldehyde-based adhesives. In addition, adhesion promoters are indicated for bonding lignocellulosic particles and polymers [6,8,14,26,27]. Therefore, based on the literature data and our previous studies, we decided to apply pMDI in the production of boards [11,36,37].

The material intended for the external layers was dried up to 2% humidity, and that for the core layer was up to 9%. The material was dried in a laboratory drum dryer (DAN LAB, Białystok, Poland). The moisture distribution is not typical for particleboards because the outer layers had much lower humidity than the middle layer. However, good results of combining thermoplastic polymers with lignocellulosic particles are obtained without water content in the plant material. On the other hand, pMDI itself requires higher humidity than the assumed 2%. It was assumed, however, that a lower straw moisture content than recommended in industrial practice for pMDI crosslinking can be compensated for by better anchoring the polymers [9,11].

The proportion of external and core layers was 3:7. The pressing conditions are presented in Table 2.

The outer layers needed to be overheated to allow for the glue’s crosslinking inside the board. The pressing time was more than doubled.

### 2.3. Board Testing

After the boards were conditioned for seven days at 21 ± 1 °C and relative air humidity of 55 ± 5%, the produced boards were tested in terms of the following parameters, according to the relevant standards:

- Bending strength (MOR) and modulus of elasticity (MOE) according to EN 310 [22];
- Internal bond (IB) according to EN 319 [42];
- Internal bond after the boiling test (V-100) according to EN-1087-1 [43];
- Thickness swelling (TS) after 24 h according to EN 317 and water absorption (WA) [44].

The investigations of water resistance and mechanical properties involved 10-to-16 samples in each variant. The number of samples was in accordance with the relevant standards indicated above.

The quality of the melted polymers and their interaction with straw particles were also analyzed using computed tomography. For this purpose, samples from boards made from triticale straw were scanned. Scanning was performed with a Hyperion X9Pro CT scanner with a resolution of 0.15 mm, at a tube voltage of 90 kV, a point resolution of 68 m, and a maximum imaging field of 13 cm × 16 cm (MyRay, Via Bicocca, Imola, Bologna, Italy). The Hounsfield (HU) scale was used to estimate the particleboard structure. The Hounsfield unit is a relative quantitative measurement of radio frequency density used by radiologists to interpret computed tomography (CT) images. Although this is not a very accurate measure, it allows for determining changes in the structure of the particleboard.

### 2.4. Attenuated Total Reflection–Fourier-Transform Infrared Spectroscopy (ATR-FTIR)

The spectra of tested boards were recorded by a Nicolet iS5 spectrophotometer (Thermo Fisher Scientific, Waltham, MA, USA) with Fourier transform, a deuterated triglycine sulfate (DTGS) detector, and an attenuated total reflection (ATR) attachment. The spectra were recorded over the range of 4000–500 cm^−1^, at a resolution of 4 cm^−1^ and with 32 co-added scans. Ten measurements for each sample were recorded by re-sampling at different locations across the entire sample.

### 2.5. Thermogravimetric Analysis (TGA)

The thermogravimetric analysis (TGA) of boards was evaluated using a Netzsch STA 449 F5 Jupiter apparatus (Erich NETZSCH GmbH and Co. Holding KG, Selb, Germany). The board samples of 20 ± 1 mg were heated at the rate of 5 °C/min to the assumed final temperature of 600 °C in the atmosphere of helium flowing through the furnace space at a rate of 15 mL/min. Thermogravimetric (TG) curves and differential thermogravimetric (DTG) curves were recorded on the thermograms.

### 2.6. Statistical Analysis

The results were analyzed using STATISTICA 13.0 package (StatSoft Inc., Tulsa, OK, USA). The performed analysis was based on ANOVA (analysis of variance), and homogeneous groups were distinguished with the use of Tukey’s HSD (honestly significant difference) test. Homogeneous groups are marked with lowercase letters. The results were analyzed at a significance level of *p* = 0.05.

## 3. Results and Discussion

**Figure 1:**
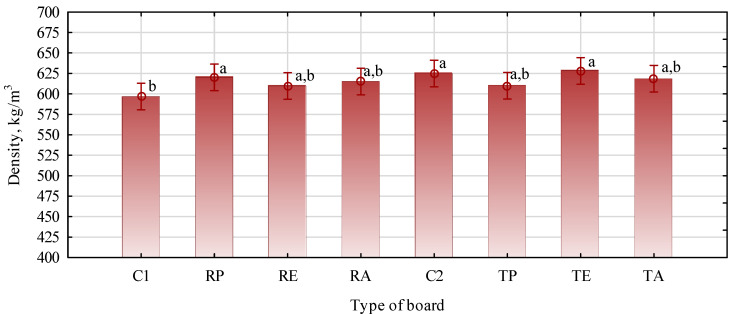
Densities of the produced boards: C1, control with rye straw; C2, control with triticale straw; R, rye straw; T, triticale straw; P, PP; E, HDPE; A, PLA. Values denoted with identical letters do not differ significantly in LSD test, F(7, 88) = 1.4982, *p* = 0.017834.

The critical feature related to the mechanical properties of wood-based materials is a strong relation with density. In the manufacturing process, it was assumed that the density of the dry boards would be 575 kg/m^3^ at a moisture content of 6.2 (SD = 0.39). Their density should be about 610 kg/m^3^ during mechanical tests. As seen in Figure 1, the data show that the boards had a density close to the established one; however, according to the ANOVA statistical analysis, the densities of individual variants differed. The difference between the variants did not exceed 30 kg/m^3^. The similar density of individual samples and, thus, the low SD (standard deviation) values, indicated statistically significant differences. The so-called conservative LSD (Least Significant Difference) test confirmed this, the results of which are also shown in Figure 1. The analysis using a less conservative post hoc test, which is more common in this type of analysis, i.e., Tuckey’s HSD, did not show statistically significant differences in the density of individual types of boards. This is important because it is easier to analyze the behavior of the boards during the tests of their mechanical properties, as well as swelling or water absorption.

**Figure 2:**
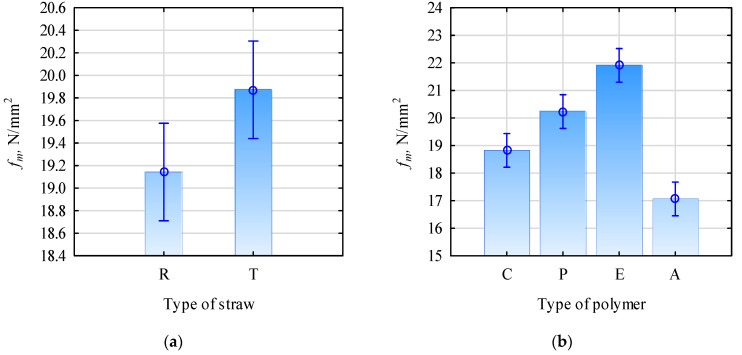
(**a**) Two-way-ANOVA effect of straw type, F(1, 88) = 5.6101, *p* = 0.02005. (Whiskers indicate 0.95 confidence intervals.) (**b**) Two-way-ANOVA effect of polymer type, F(3, 88)=44.850, *p* = 0.0000. (Whiskers indicate 0.95 confidence intervals.) C, control; R, rye straw; T, triticale straw; P, PP; E, HDPE; A, PLA.

As expected, based on previous studies [11] the mechanical properties of boards made of triticale straw particles were slightly higher than boards made of rye straw particles. In addition, good mechanical properties were observed for single-layer boards made from rape particles [45]. This was applied primarily to the bending strength and modulus of elasticity. In the case of the static bending strength, a variance ANOVA was analyzed. It showed that boards made of triticale straw were characterized by a higher static bending strength than boards made of rye straw particles (Figure 2a). However, from a technological point of view, these differences were insignificant, as they did not exceed 4%. The type of polymer used affects the static bending strength of the manufactured boards more (Figure 2b). In this case, we observed an apparent increase in the strength of boards made with HDPE and a noticeable decrease in the strength of boards made with PLA. These changes were at +15% in the case of boards with HDPE, and −10% with PLA.

**Figure 3:**
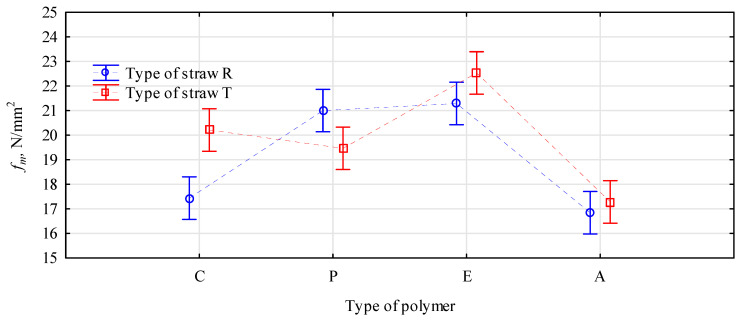
Graph of the interaction of the influence of the type of polymer and the type of straw on the bending strength, F(3, 88) = 8.4941, *p* = 0.00005. C, control; P, PP; E, HDPE; A, PLA.

**Figure 4:**
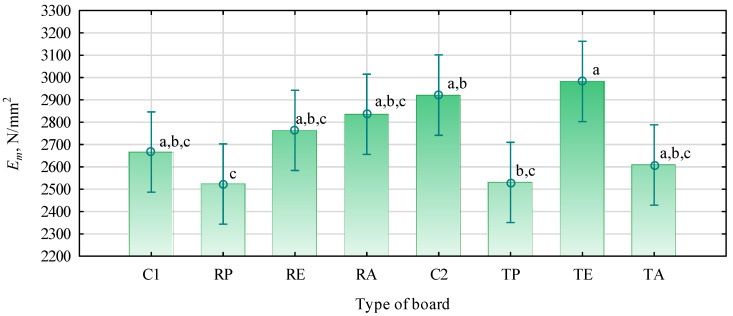
Modulus of elasticity of the produced boards: C1, control with rye straw; C2, control with triticale straw; R, rye straw; T, triticale straw; P, PP; E, HDPE; A, PLA. Values denoted with identical letters do not differ significantly in HSD Tukey’s test, F(7, 88) = 3.7348, *p =* 0.00138.

The statistical analysis of the interaction between the type of straw and the polymer shows no such relation (Figure 3). There were no statistically confirmed premises to recognize that the static bending strength of straw boards with the addition of polymers is related to the type of straw. Therefore, it can be assumed that some polymers work better with a given straw and others work worse. The data presented in Figure 3 indicate that only boards made of triticale with the addition of PP showed a lower bending strength than those based on rye straw. Interestingly, these boards had a very low modulus of elasticity (Figure 4). Their modulus of elasticity was more than 2500 N/mm^2^, which can be considered high. The boards of wood chips of a similar thickness and density had a modulus of elasticity of 2200 N/mm^2^. However, compared to the C2 control board, the modulus of elasticity of the TP-type board was nearly 15% lower. The changes in modulus of elasticity boards with polymers were also observed in other studies [36,37,46,47].

The modulus of elasticity of the boards produced in the proposed manner was relatively untypical and deviated from expectations. The board with polymers of a relatively high strength showed a lower modulus of elasticity than those with lower strength. The exception was TE-type boards, i.e., with external layers made of triticale straw and HDPE. In their case, a high bending strength was correlated with high stiffness. There may be a reduction in the stiffness of straw boards produced with thermoplastic polymers since they naturally have lower stiffness. However, boards based on straw particles or wood, made with about 20% polymers, do not have to behave like typical polymer composites. In the case of the analyzed three-layer boards, the quality of the outer layers determines the stiffness. It depends on the quality of the polymer–straw bond, the distribution of polymers in the layer structure, the degree of compaction, and its structure. It should be recognized that the stiffness of straw–polymer boards determined by the modulus of elasticity is most favorably influenced by HDPE, and it is the least favorably influenced by PP. Both polymers originated from packaging crushing and had a similar form after grinding. Straw particles, unlike wood, do not have a good structure for anchoring thermoplastic polymers. In the case of boards made of pine chips and thermoplastic polymers, Borysiuk [34] obtained good results. However, the specialized treatments suitable for wood were different from straw. The smooth surface of straws from the grass genus and the spongy straw from the cabbage family do not create places for the mechanical embedding of molten polymers. In their case, the agents acting as adhesion promoters, the appropriate degree of fragmentation of both materials, and the technological parameters of producing such boards play an essential role.

**Figure 5:**
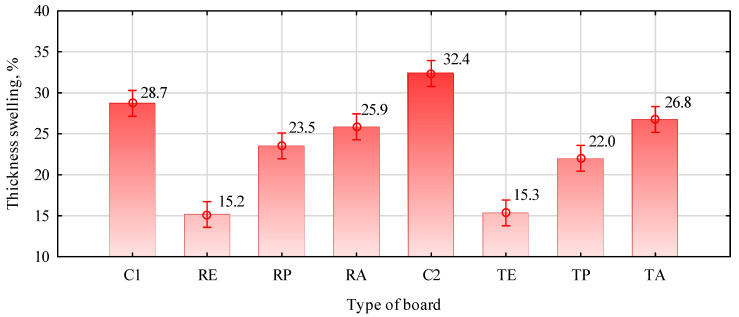
The effect of thermoplastic polymers on the swelling of the thickness of the boards after 24 h of soaking in water, F(7, 96) = 59.034, *p* = 0.0000. C1, control with rye straw; C2, control with triticale straw; R, rye straw; T, triticale straw; P, PP; E, HDPE; A, PLA.

The fulfillment of the first two factors may be evidenced by the swelling of the boards to the thickness after soaking in water. If the lignocellulosic material and thermoplastic polymer are properly and permanently merged, then the swelling of the boards produced in this way should be lower. The data presented in Figure 5 show that all boards with the addition of thermoplastic polymers to the outer layers had lower swelling than the control boards. The swelling of the control boards was about 31%, while that of the straw–polymer boards was about 21%. Generally, a positive effect is obtained when the swelling of the straw–polymer boards is lower than 24%. This condition still needed to be met by boards with PLA, and it only looked slightly better in the case of boards with PP and triticale straws. On the other hand, in the case of boards with HDPE, the swelling of boards, regardless of the type of straw, slightly exceeded 15%, which is about 50% lower than that of the control boards.

**Figure 6:**
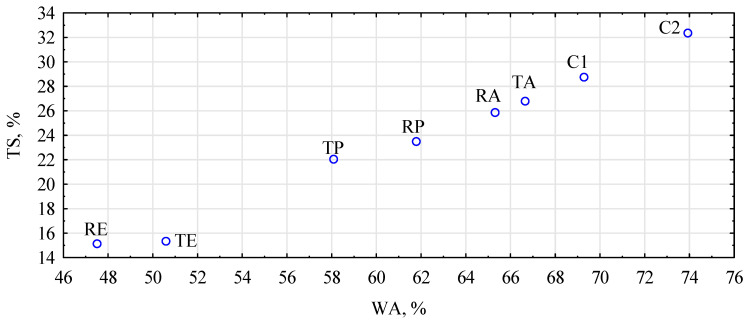
The dependence of board swelling on their water absorption after 24 h of soaking in water. C1, control with rye straw; C2, control with triticale straw; R, rye straw; T, triticale straw; P, PP; E, HDPE; A, PLA.

The swelling determined after 24 h of soaking in water is correlated with water absorption (Figure 6). Of course, the more water the board absorbs, the more intensively it swells; however, to absorb water intensively, it must have favorable conditions. On the one hand, there is high porosity, and on the other, there is easy access to the lignocellulosic structure. Thus, assuming that the boards were of a similar density, the possibility of penetration was similar. In contrast, with better coverage of the chopped straw with polymers and better compactness due to board gluing, its susceptibility to water absorption inside the lignocellulosic material was lower. The higher resistance to water absorption was also observed for the wood–plastic composite that was flat-pressed [48]. Generally, it can be assumed that the addition of polymers improves the water resistance of particleboard regardless of lignocellulose particles.

**Figure 7:**
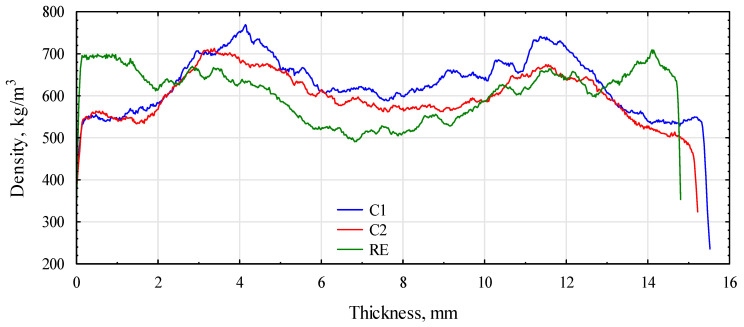
Board density profiles based on triticale and rye HDPE. C1, control with rye straw; C2, control with triticale straw; RE, rye with HDPE.

**Figure 8:**
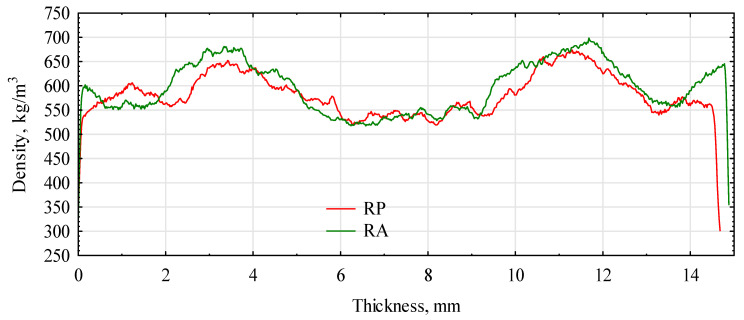
Rye-based board density profiles with PP and PLA. RP, rye straw/PP; RA, rye straw/PLA.

The assumed board-manufacturing conditions and the type of polymer significantly influenced the formation of the density profile of the produced boards. Figure 7 and Figure 8 show the density profiles for boards made of triticale and rye particles.

Both profiles had a similar shape. Due to the low humidity of the material used to form the outer layers, the zones of maximum density were very strongly shifted into the board’s structure. They were located about 4 mm below the surface layers. The middle layer, on the other hand, was compacted to a density of more than 600 kg/m^3^. With a low density of near-surface layers, it had lower properties determined in the bend test. The density profile for the RE board looked the opposite way.

**Figure 9:**
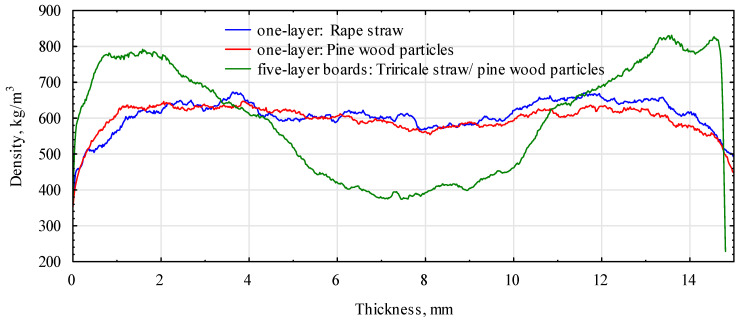
Exemplary density profiles of boards based on straw and wood particles.

In this case, the layers with the highest density were located in the zone of about 1.75 mm from the surface, and the middle layer, from about 6 mm from the surface, had a density close to 500 kg/m^3^. Interestingly, the PLA boards had slightly compacted layers at the very surface and areas of maximum density of about 3 mm below the surface. This contributed to the high modulus of elasticity of these boards. The TA boards (triticale with PLA) confirmed this with a profile similar to the control board. Boards with the addition of PP had an intermediate density profile shape. The shape of the profile for any variant was unsymmetrical because the boards were produced in a shelf press. The side on the hot shelf heats up much faster than the top side, which starts heating a few seconds later. In continuous presses, such an effect should not occur. The modification consisting of the production of boards from two species of grass with different bulk densities did not fully bring the expected result. There was some compaction of the outer layers, but it was not as intense as expected. Boards produced as a single layer from the same material under similar conditions had a very flat density profile (Figure 9). Nearly 80% of the thickness of the board had a density of approximately 600 kg/m^3^. This adversely affected the static bending strength and, above all, the modulus of elasticity. The higher density of the near-surface layers also delays the thickness swelling of the boards. The introduction of thermoplastic polymers to the outer layers increased the density of these layers; however, an apparent effect was noticed only in the case of RE or RP boards.

**Figure 10:**
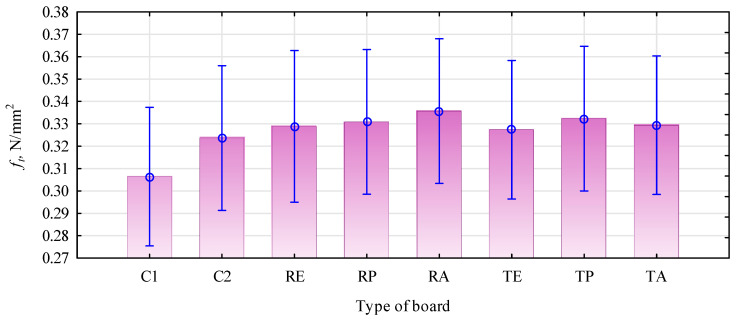
Tensile strength perpendicular to the surfaces of boards based on straw and thermoplastic polymers. C1, control with rye straw; C2, control with triticale straw; R, rye straw; T, triticale straw; P, PP; E, HDPE; A, PLA.

However, this was a minor increase compared to boards manufactured as five layers. However, producing three-layer boards with distinctly different layers is much easier than five-layer boards. The shape of the density profile did not significantly affect the tensile strength perpendicular to the surface test (Figure 10).

The obtained results ranged from 0.31 N/mm^2^ to 0.33 N/mm^2^, which was a value close to that often found for this type of plate produced in a laboratory. The samples were destroyed in the middle layer, made of the same straw, and glued similarly; the results should be similar.

Since adding polymers changed the density profile, it could change the damage site to the outer layers. Such an effect, i.e., tearing of the samples in the outer layer, could also have occurred due to the degradation of this layer in the pressing process (wrongly selected pressing conditions and/or incorrect binder is chosen). According to the presented data, the tensile strength perpendicular to the surface test of the analyzed boards did not differ from the previously analyzed rape-based boards of the same density [36]. It was lower by about 20% than the strength of boards with a density of 625 kg/m^3^ [11].

Well-prepared pressing conditions allow for the creation of a layer in the outer layer of the boards that blocks water migration into the board. In previous studies, we have shown that when protecting the side surfaces, water penetrated very slowly into the board through the surfaces containing polymers, causing a slight increase in the thickness of the board [31]. Supplementary Figures S1–S3 show the appearance of the surface and its analysis using computed tomography. On the selected cross-sections, the density distribution was determined along the baseline (the green line), while the red line marks the place on the cross-section.

The data in Supplementary Figure S1 refer to the TE board and the structural analysis in the near-surface layer, i.e., about 1 mm below the sample’s surface. The surface was covered with a polymer; it was smooth and quite bright. The radiological density defined on the Hounsfield scale ranges from −336 to 336 HU.

The negative value the red point on the profile, is placed in the dark area associated with the surface of the straw, while the positive value is in the white area, which is related to the HDPE. Supplementary Materials Figure S3 shows the profile for the sample’s surface in the middle of the board’s thickness. The histogram has shown changes from -589 to 25 HU, with no clear high positive values. Air density under standard conditions was defined as −1000 HU, although it was generally assumed to be below −700 HU. The radiation density for wood with a 600–800 kg/m^3^ is −400–200 HU [44]. Thus, strands of straw and free spaces dominated the central zone.

**Figure 11:**
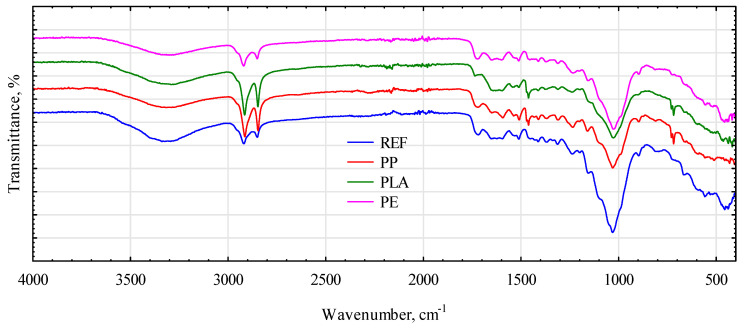
The spectra of particleboards with rye straw. REF, rye straw without polymer; PP, polypropylene; PLA, polylactic acid; PE, high-density polyethylene.

In contrast, in the outer zone, in which thermoplastic polymers had bonded with straw chopped, the radiation density value significantly increased above 0 HU. It was confirmed by the radiation density profile on the control sample (board C2), at a similar depth, in the near-surface layer. Similar to the analysis of the middle layer, it was significantly below 0 HU, which indicates the presence of lignocellulosic material with a certain degree of density. The structure of the analyzed straw-based boards was determined by infrared spectroscopy, and the results are presented as spectra in Figure 11. The ATR-FTIR spectra of particleboards made from rye straw contained several well-defined peaks that are characteristic of lignocellulosic materials. The presented spectra were interpreted based on the available literature data [49,50,51,52,53].

**Figure 12:**
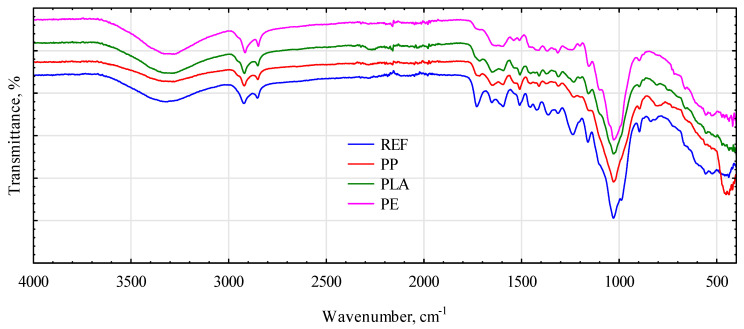
The spectra of particleboards with triticale straw. REF, triticale straw without polymer; PP, polypropylene; PLA, polylactic acid; PE, high-density polyethylene.

The broad peak between 3600 and 3200 cm^−1^ corresponds to the O-H stretching of water associated with the intermolecular hydrogen bond. The spectrum of the particleboard sample without a polymer matrix showed a higher intensity of this band, which is related to the hydrophilic tendency of lignocellulosic cereal material. The spectra of all samples contain peaks at approximately 2920 cm^−1^ and 2850 cm^−1^, arising from symmetric and asymmetric stretching vibrations in methoxyl, methyl, and methylene groups in the structure of saccharides and aromatic compounds. The peak at approximately 1720 cm^−1^ observed in the IR spectra of all samples can be assigned to the C=O stretching of the acetyl ester group of hemicellulose and/or the ester linkage of the carboxylic group of the ferulic and coumaric acids of lignin. The bands in the range of 1600–1500 cm^−1^ can represent the aromatic ring stretch, including the C=C bond of aromatic skeletal vibrations in lignin. The peak at 1460 cm^−1^ can be attributed to the C-H deformation stretching in lignin and xylan. In turn, the 1200–1000 cm^−1^ region represents the C-O stretching and deformation bands in cellulose and lignin, and the bands in the 900–690 cm^−1^ range can be described as C-H bending. In addition, overlapping peaks from the straw and polymer matrix can be observed in the spectra of the polymer-matrix particleboard [54,55,56]. Similar peaks to those in the particleboard with rye were observed in the spectra of samples with triticale (Figure 12). However, these peaks differ in the intensity of the transmittance, related to the different amounts of characteristic groups (varied content of the main components: cellulose, lignin, and hemicellulose). Moreover, in some cases, the peaks for the same constituent were shifted, which is associated with the nature of hydrogen bonding and coupling effects.

**Figure 13:**
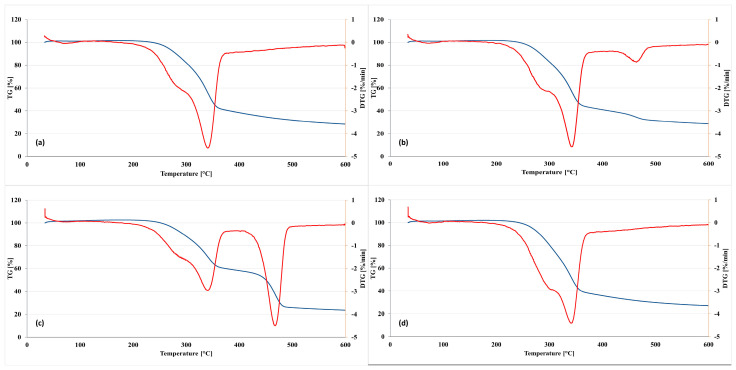
TGA (blue line) and DTG (red line) curves of triticale-based boards: (**a**) control, (**b**) TP, (**c**) TE, and (**d**) TA. T, triticale straw; P, PP; E, HDPE; A, PLA.

The weight losses of triticale-based board samples in relation to the temperature of thermal degradation (TG curve) and the first derivative of that curve (DTG) are presented in Figure 13.

The DTG curve for the sample prepared from triticale without a polymer matrix (Figure 13a) showed a single decomposition step, and the decomposition peak temperature (T_max_) was 341 °C, which is in agreement with the literature data [38,57]. The boards consisted of triticale, and PLA (Figure 13d) also degraded in one step, just like the samples from the lignocellulosic material alone. The T_max_ of the PLA-triticale composite was observed at the same temperature as for the biomass sample alone and was 341 °C. According to the literature data, the T_max_ for pure PLA is around 362 °C [40]. Therefore, the overlap of decomposition peaks can be observed in this case. Adding another polymer matrix (PP and HDPE) to the biomass caused a change in the thermal behavior of the obtained composites. The boards with PP and HDPE curves (Figure 13b,c) presented two steps for weight loss. The first weight loss of the triticale-PP composite, which can be related to biomass degradation, started at 342 °C. The second weight loss of this composite was at 464 °C and can be connected with the degradation of PP, for which the T_max_, according to the literature data, is around 455 °C [39]. The two peaks were also observed on the DTG curve of the triticale-HDPE composite, as shown in Figure 13c. The minor degradation peak temperature (T_max1_) at 340 °C was related to the straw degradation, and the higher degradation peak temperature (T_max2_) at 467 °C resulted from the degradation of polyethylene. The onset of all composites degradation (T_onset_) began at 168–189 °C depending on the composite type. According to the literature reports, the thermal degradation of hemicellulose occurs in the temperature range of 150–315 °C, while the depolymerization of cellulose takes place between 275 and 400 °C. In turn, the decomposition of lignin covers a broad temperature range of 150–500 °C [40,41,57]. All composite samples showed a mass loss between 69.59% (triticale composite) and 76.67% (triticale-HDPE composite) upon reaching 500 °C.

## 4. Conclusions

The properties of boards based on lignocellulose particles, referred to as chips, strips, or chopped strands, depend on several technological factors that have been known for years. However, there is still a need for research to refine specific solutions, especially when trying to add new materials to the production of boards. Based on the result, the following conclusions were drawn:

- There is a possibility of producing lignocellulosic boards from two different straws, the less advantageous of which should be the middle layer of these boards. Because the straws of grass origin stick together more efficiently and form more compact layers, using them in outer layers is better. Rape straws, with a completely different structure, can be successfully used as the middle layer of such boards. The rapeseed straw, with different structures, allows for good shaping of the middle layer’s structure, despite the parenchyma tissue’s negative impact on the gluing process. However, the research in this area can be continued by even greater diversification of the bulk density or moisture content of the layers;
- Introducing thermoplastics only to the outer layers is beneficial due to the more straightforward melting process. It affected the boards’ properties determined in the bend test by affecting the density profile. It can be concluded that the beneficial effect of thermoplastics on the board density profile masks their low elastic properties;
- Thermoplastics, which were successfully merged with the lignocellulosic material, significantly improved the hydrophobic properties of the boards by reducing their swelling in thickness and reducing water absorption;
- Among the straw-based boards with the addition of tested polymers, satisfactory properties were produced mainly using HDPE, and moderate properties using PP. PLA-containing boards did not show clearly favorable properties either in terms of mechanical or physical features;
- The properties of straw–polymer boards produced based on triticale straw were slightly better than rye-based boards, probably due to the geometry of the strands, which was more favorable for triticale straw;
- The particleboards made from straw with the addition of polymers due to lower hygroscopicity and thickness swelling compared to traditional boards can be used in conditions of increased humidity, i.e., garden, bathroom, or kitchen furniture;
- The study should continue to further develop the finishing process.

## Figures and Tables

**Table 1:** Structure of polymer-enriched boards.

Symbol	External Layers	Core Layer	Content of Polymer	Polymer
Control 1 (reference sample)	Rye straw	Rape straw	-	-
RP	Rye straw	Rape straw	30%	PP
RE	Rye straw	Rape straw	30%	HDPE
RA	Rye straw	Rape straw	30%	PLA
Control 2 (reference sample)	Triticale straw	Rape straw	-	-
TP	Triticale straw	Rape straw	30%	PP
TE	Triticale straw	Rape straw	30%	HDPE
TA	Triticale straw	Rape straw	30%	PLA

**Table 2:** Pressing conditions.

Parameter	Value
The temperature of hotplates	205 °C
Maximum pressure	2.0 N/mm^2^
Pressing time	40 s/mm of board thickness
Board thickness	15 mm
Density	575 kg/m^3^

## Data Availability

Not applicable.

## Supplementary Materials

The following supporting information can be downloaded at: https://www.mdpi.com/article/10.3390/ma16124400/s1, Figure S1. Photos and profile of the density distribution in the HU scale—the analyzed layer at the depth of the red line, the density histogram defined along the green line—TE sample. Figure S2. Photos and profile of the density distribution in the HU scale—the analyzed layer at the depth of the red line (the middle of the sample thickness), the histogram of the density along the green line—the TE sample. Figure S3. Photos and histogram of the density distribution in the HU scale—the analyzed layer at the depth of the red line (the middle of the sample thickness), the histogram of the density along the green line—sample C2.

## References

[1] Mirski R., Kawalerczyk J., Dziurka D., Wieruszewski M., Trociński A. (2020). Effects of using bark particles with various dimensions as a filler for urea-formaldehyde resin in plywood. BioResources.

[2] Gradziuk P., Gradziuk B., Trocewicz A., Jendrzejewski B. (2020). Potential of straw for energy purposes in Poland—Forecasts based on trend and causal models. Energies.

[3] Gradziuk P. (2014). The potential of straw for energy purposes in Poland. Barom. Reg..

[4] Zhang Y., Lu X., Pizzi A., Delmotte L. (2003). Wheat straw particleboard bonding improvements by enzyme pretreatment. Holz Als Roh Werkst..

[5] Boquillon N., Elbez G., Schönfeld U. (2004). Properties of wheat straw particleboards bonded with different types of resin. J. Wood Sci..

[6] Bekhta P., Korkut S., Hiziroglu S. (2013). Effect of pretreatment of raw materials on properties of particleboards panels made from wheat straw. BioResources.

[7] Azizi K., Tabarsa T., Ashori A. (2011). Performance characterizations of particleboards made with wheat straw and waste veneer splinters. Compos. B. Eng..

[8] Mo X., Cheng E., Wang D., Sun X.S. (2003). Physical properties of medium-density wheat straw particleboard using different adhesives. Ind. Crops Prod..

[9] Dziurka D., Mirski R. (2013). Lightweight boards from wood and rape straw particles. Drewno.

[10] Huang L., Xia P., Liu Y., Fu Y., Jiang Y., Liu S., Wang X. (2016). Production of biodegradable boards using rape straw and analysis of mechanical properties. BioResources.

[11] Mirski R., Dziurka D., Banaszak A. (2018). Properties of particleboards produced from various lignocellulosic particles. BioResources.

[12] Li X., Cai Z., Winandy J.E., Basta A.H. (2010). Selected properties of particleboard panels manufactured from rice straws of different geometries. Bioresour. Technol..

[13] Zhang L., Hu Y. (2014). Novel lignocellulosic hybrid particleboard composites made from rice straws and coir fibers. Mater. Des..

[14] Kurochi Y., Sato M. (2015). Effect of surface structure, wax silica on the properties of binderless board made from rice straw. Ind. Crop Prod..

[15] Wu T., Wang X., Kito K. (2015). Effects of pressures on the mechanical properties of corn straw bio-boards. Eng. Agri. Environ. Food.

[16] Akinyemi B.A., Kolajo T.E., Adedolu O. (2022). Blended formaldehyde adhesive bonded particleboards made from groundnut shell and rice husk wastes. Clean Technol. Environ. Policy.

[17] Dukarska D., Bartkowiak M., Stachowiak-Wencek A. (2015). White mustard straw as an alternative raw materials in manufacturing particleboards resinated with different amounts of urea formaldehyde resin. Drewno.

[18] Dukarska D., Łęcka J., Czarnecki R. (2015). The effect of wood chip substitution with evening primrose waste on properties of particleboards depending on the type of binding agent. EJPAU.

[19] Bekalo S.A., Reinhardt H.-W. (2010). Fibers of coffee husk and hulls for the production of particleboard. Mater. Struct..

[20] Guuntekin E., Uner B., Karakus B. (2009). Chemical composition of tomato (*Solanum lycopersicum*) stalk and suitability in the particleboard production. J. Environ. Biol..

[21] Nemli G., Kırcı H., Serdar B., Ay N. (2003). Suitability of kiwi (*Actinidia sinensis* Planch.) prunings for particleboard manufacturing. Ind. Crops Prod..

[22] (1993). Wood-Based Panels—Determination of Modulus of Elasticity in Bending and of Bending Strength.

[23] Faustino J., Pereira L., Soares S., Cruz D., Paiva P., Varum H., Ferreira J., Pinto J. (2012). Impact sound insulation technique using corn cob particleboard. Constr. Build Mater..

[24] Pinto J., Paiva A., Varum H., Costa A., Cruz D., Pereira S., Fernandes L., Tavares P., Agarwal J. (2011). Corn’s cob as a potential ecological thermal insulation material. Energ. Buildings.

[25] Pinto J., Vieira B., Pereira H., Jacinto C., Vilela P., Paiva A., Pereira S., Cunha V., Varum H. (2012). Corn cob lightweight concrete for non-structural applications. Constr. Build. Mater..

[26] Grigoriou A.H. (2000). Straw-wood composites bonded with various adhesive systems. Wood Sci. Technol..

[27] Cheng W., Han G., Fang D. (2013). Oriented structural boards from split wheat straw: Effects of straw length, panel density, and resin content. BioResources.

[28] Paukszta D., Szostak M., Rogacz M. (2014). Mechanical properties of polypropylene copolymers composites filled with rapeseed straw. Polimery.

[29] Kuciel S., Liber-Kneć A., Zajchowski S. (2010). Kompozyty z włóknami naturalnymi na osnowie recyklatu polipropylenu. Polimery.

[30] Sudhakar K., Srinivas C. (2014). Investigation of mechanical properties of rice straw fiber polypropylene composites. Int. J. Eng. Res. Appl..

[31] Mirski R., Banaszak A., Bekhta P. (2021). Selected properties of formaldehyde-free polymer-straw boards made from different types of thermoplastics and different kinds of straw. Materials.

[32] Luo P., Yang C., Li M., Wang Y. (2020). Manufacture of thin rice straw particleboards bonded with various polymeric methane diphenyl diisocyanate/ urea formaldehyde resin mixtures. BioResorces.

[33] Pawlicki J., Niecewicz D. Waste thermoplastic polymers in technologies of wood-based panels. Proceedings of the III International Symposium “Wood Agglomeration".

[34] Borysiuk P. (2012). Możliwość wytwarzania płyt wiórowo-polimerowych z wykorzystaniem poużytkowych termoplastycznych tworzyw sztucznych. Rozpr. Nauk. Monogr. Szkoła Główna Gospod. Wiej. Warszawie.

[35] Jastrząb J. (2014). Badania nad Zastosowaniem Termoplastów w Procesie Wytwarzania Płyt OSB. Ph.D. Thesis.

[36] Mirski R., Dziurka D., Banaszak A. (2019). Effects of manufacture conditions on physical and mechanical properties of rape-polymers board. Wood Res..

[37] Mirski R., Dziurka D., Banaszak A. (2019). Using rape particles in the production of polymer and lignocellulose boards. BioResources.

[38] Mihai M., Ton-That M.-T. (2018). Valorization of triticale straw biomass as reinforcement in proficient polypropylene biocomposites. Waste Biomass. Valor..

[39] Mihai M., Ton-That M.-T., Ngo T.-D., Busnel F. New polypropylene/triticale composites: Relationship between formulation and properties. Proceedings of the 69th Annual Technical Conference of the Society of Plastics Engineers 2011 (ANTEC 2011).

[40] Mihai M., Ton-That M.-T. (2014). Novel polyactide/triticale straw biocomposites: Processing, formulation, and properties. Polym. Eng. Sci..

[41] Mamun A.A., Heim H.P., Bledzki A.K. (2015). The use of maize, oat, barley and rye fibers as reinforcements in composites. Biofiber Reinforcements in Composites Materials.

[42] (1993). Particleboards and Fibreboards—Determination of Tensile Strength Perpendicular to the Plane of the Board.

[43] (1995). Particleboards—Determination of Moisture Resistance—Boil Test.

[44] (1993). Particleboards and Fibreboards. Determination of Swelling in Thickness after Immersion in Water.

[45] Dukarska D., Czarnecki R., Dziurka D., Mirski R. (2017). Construction particleboards made from rapeseed straw glued with hybrid pMDI/PF resin. Eur. J. Wood Wood Prod..

[46] Borysiak S., Paukszta D. (2008). Mechanical properties of lignocellulosic/polypropylene composites. Mol. Cryst. Liq. Cryst..

[47] Borysiuk P., Mamiński M., Zado A. (2009). Some comments on the manufacturing of thermoplastic-bonded particleboards. Ann. Wars. Univ. Life Sci. For. Wood Technol..

[48] Borysiuk P., Wilkowski J., Krajewski K., Auriga R., Skomorucha A., Auriga A. (2020). Selected properties of flat-pressed wood-polymer composites for high humidity condition. BioResources.

[49] Bledzki A.K., Mamun A.A., Volk J. (2010). Physical, chemical and surface properties of wheat husk, rye husk and soft wood and their polypropylene composites. Compos. Part A.

[50] Sun R.C., Sun X.F. (2002). Structural and thermal characterization of acetylated rice, wheat, rye, and barley straws and poplar wood fibre. Ind. Crops Prod..

[51] Kalina M., Sovova S., Svec J., Trudicova M., Hajzler J., Kubikova L., Enev V. (2022). The effect of pyrolysis temperature and the source biomass on the properties of biochar produced for the agronomical applications as the soil conditioner. Materials.

[52] Vitolina S., Shulga G., Neiberte G., Jaunslavietis J., Verovkins A., Betkers T. (2022). Characteristics of the waste wood biomass and its effect on the properties of wood sanding dust/recycled PP composite. Polymers.

[53] Woźniak M., Ratajczak I., Wojcieszak D., Waśkiewicz A., Szentner K., Przybył J., Borysiak S., Goliński P. (2021). Chemical and structural characterization of maize stover fractions in aspect of its possible applications. Materials.

[54] Luna I.Z., Dam K.C., Chowdhury S., Gafur A., Khan N., Khan R.A. (2015). Physical and thermal characterization of alkali treated rice husk reinforced polypropylene composites. Adv. Mater. Sci. Eng..

[55] Mofokeng J.P., Luyt A.S., Tabi T., Kovacs J. (2012). Comparison of injection moulded, natural fibre-reinforced composites with PP and PLA as matrices. J. Thermoplast. Compos. Mater..

[56] Singla P., Mehta R., Berek D., Upadhyay S.N. (2012). Microwave assisted synthesis of poly(lactic acid) and its characterization using size exclusion chromatography. J. Macromol. Sci..

[57] Ross K., Godfrey D. (2012). Effect of extractives on the thermal decomposition of wheat, triticale and flax crop residues: A kinetic study. IJBR.

