# Supplementary material for: Annual Plants and Thermoplastics in the Production of Polymer and Lignocellulose Boards
Source: Materials (Basel). 2023 Jun 15;16(12):4400. doi: 10.3390/ma16124400 (PMC10304041; doi:10.3390/ma16124400)
Supplement: Supplementary file 1 [file materials-16-04400-s001.zip › materials-2418641-supplementary.pdf]

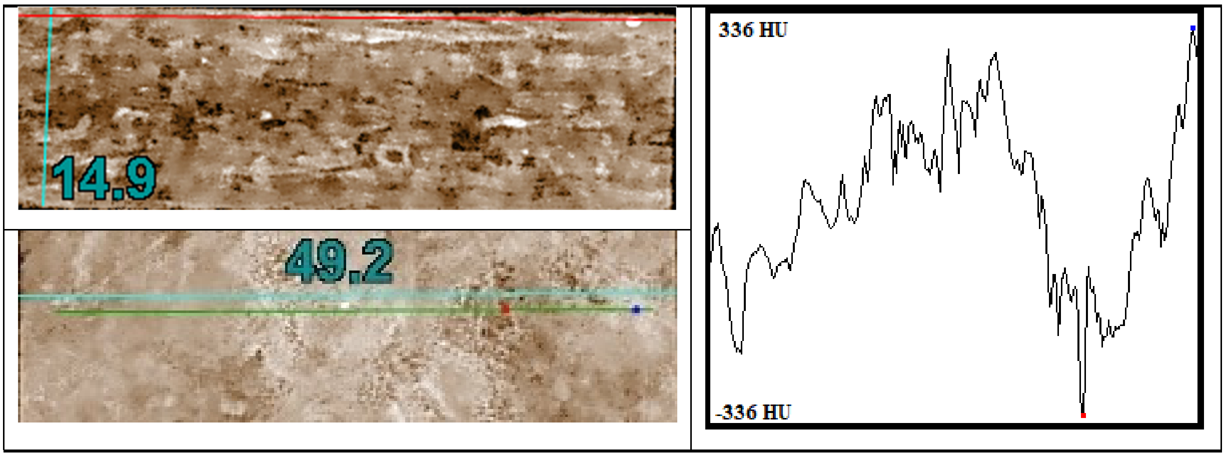

Figure S1. Photos and profile of the density distribution in the HU scale – the analyzed layer at the depth of the red line, the density histogram defined along the green line – TE sample.

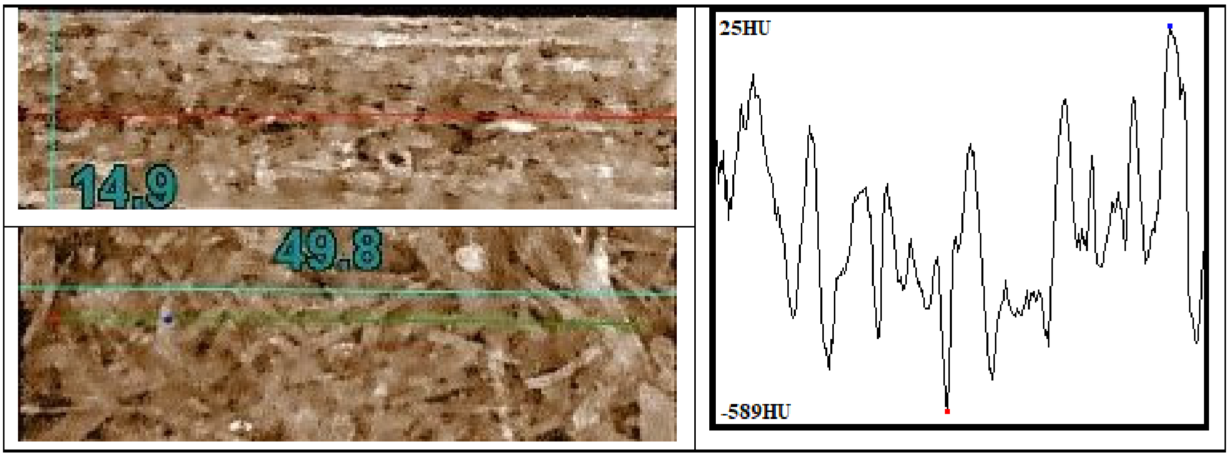

Figure S2. Photos and profile of the density distribution in the HU scale - the analyzed layer at the depth of the red line (the middle of the sample thickness), the histogram of the density along the green line – the TE sample.

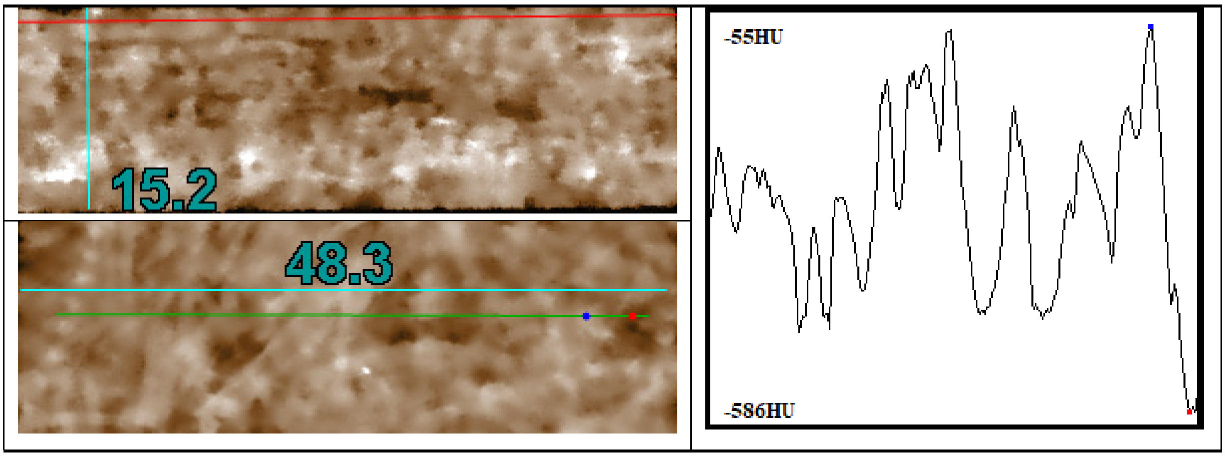

Figure S3. Photos and histogram of the density distribution in the HU scale - the analyzed layer at the depth of the red line (the middle of the sample thickness), the histogram of the density along the green line – sample C2.
